# An Evolving Role for the Long Non-Coding RNA H19 in Aging and Senescence

**DOI:** 10.1093/geroni/igab046.2153

**Published:** 2021-12-17

**Authors:** Manali Potnis, Christian Sell

**Affiliations:** Drexel University, Philadelphia, Pennsylvania, United States

## Abstract

The long non-coding RNA (lncRNA) H19 is a maternally imprinted gene transcript that, in conjunction with the neighboring Igf2 gene, is critical in controlling embryonic growth. Loss of H19 results in fetal overgrowth associated with Beckwith Weidemann syndrome, while elevated H19 occurs in human cancers. In the adult, H19 functions in cancer cells where it promotes migration and is correlated with poor prognosis, and in adult stem cells where it is a key regulator of cell fate decisions during differentiation. While the function of H19 in primary somatic cells has not been defined, a reduction in the abundance of H19 has been reported during senescence in endothelial cells. Given the critical importance of H19 in cell fate decisions, it is likely that understanding the precise function of H19 in somatic cells in general and why reduced levels occur with cellular senescence will provide novel insights into both somatic cell maintenance and the senescence program. Towards this end, we examined the role of H19 in somatic cell growth using cardiac interstitial fibroblasts. Our results indicate that H19 is not only vital for somatic cell proliferation and survival, but that depletion of H19 leads to cell cycle arrest and the formation of abnormal nuclei resulting in senescent cells. We are defining both the upstream regulators of H19 and the downstream mediators of senescence following H19 depletion. Overall, these results indicate an essential role for H19 in cell cycle progression, chromatin structure, and possibly proper mitotic division.

